# The Impact of Real-World Alternative Dosing Strategies of Palbociclib on Progression-Free Survival in Patients with Metastatic Breast Cancer

**DOI:** 10.3390/curroncol29030145

**Published:** 2022-03-07

**Authors:** Fulbert Fu, Jessica Kano, Julia Ma, Mera Guindy

**Affiliations:** 1 Trillium Health Partners, Mississauga, ON L5M 2N1, Canada; jessica.kano@thp.ca (J.K.); mguindy@mohawkmedbuy.ca (M.G.); 2 Institute for Better Health, Trillium Health Partners, Mississauga, ON L5M 2N1, Canada; julia@precision-analytics.ca; 3 Mohawk Medbuy Corporation, Burlington, ON L7L 0A1, Canada

**Keywords:** palbociclib, letrozole, fulvestrant, metastatic breast cancer, alternative dosing strategies, progression-free survival, neutropenia

## Abstract

Background: Palbociclib, a cyclin-dependent kinase 4 and 6 (CDK 4/6) inhibitor, in combination with letrozole or fulvestrant has been demonstrated to prolong the progression-free survival (PFS) in patients with hormone receptor-positive (HR+), human epidermal growth factor 2-negative (HER2-) metastatic breast cancer. In efforts to mitigate neutropenic toxicities, oncologists in real-world practice have prescribed alternative dosing strategies with palbociclib, yet the implication on PFS is unknown. Methods: We conducted a retrospective, observational chart review of all female patients at our clinics with HR+, HER2- metastatic breast cancer receiving palbociclib in combination with either letrozole or fulvestrant with a first dose initiated between June 2016 and December 2018 and followed their disease course until 30 April 2020. Results: The median PFS for all clinic patients receiving palbociclib and letrozole (*n* = 63) was 40.8 months (95% confidence interval (CI) 25.6–not estimable) and 16.97 months (95% CI 8.57–not estimable) for patients receiving palbociclib and fulvestrant (*n* = 11). We identified seven alternative dosing strategies prescribed by oncologists, the most prevalent being prescribing palbociclib for three weeks on and two weeks off (*n* = 8). The Kaplan–Meier curves for PFS in patients receiving letrozole and palbociclib prescribed alternative dosing strategies appear to diverge from monograph dosing early in the treatment. Many patients prescribed palbociclib using alternative dosing strategies continued to be observed even by the 18-month timepoint. The prevalence of grade 4 neutropenia was lower for patients on palbociclib with letrozole, suggesting a possible mitigation of severe neutropenia with alternative dosing strategies. Conclusions: We conclude that alternative dosing strategies used by oncologists such as prescribing palbociclib for three weeks on, two weeks off may achieve comparable disease control while mitigating neutropenic toxicities when compared to standard monograph dosing recommendations, prolonging treatment tolerability and adherence. Further large-scale studies are needed to confirm these results for future clinical adoption.

## 1. Introduction

Breast cancer is the second most diagnosed cancer worldwide and the most commonly diagnosed cancer in women [1,2]. While early breast cancer has excellent prognosis, metastatic breast cancer remains incurable with current therapies [3].

Since their discovery, hormone receptor (HR) and human epidermal growth factor 2 (HER2) have been essential biomarkers that predict disease prognosis and guide treatment decisions [4,5]. In the United States, HR-positive, HER2-negative breast cancer constitute more than 70% of cases [6]. Traditionally, endocrine therapy was the first-line treatment for metastatic HR-positive, HER2-negative breast cancers, but the development of resistance towards endocrine therapy would subsequently necessitate chemotherapy [7]. This has led research efforts towards alternative pharmacotherapy targeting cell cycle pathways.

Palbociclib is a reversible inhibitor of cyclin-dependent kinases 4 and 6 (CDK4/6) that phosphorylates the retinoblastoma protein b (Rb) and results in cell cycle arrest [8,9]. In the landmark PALOMA-2 study, the combination of palbociclib and letrozole in advanced breast cancer resulted in a median progression-free survival (PFS) of 24.8 months compared with 14.5 months in the placebo-letrozole group (hazard ratio (HR) 0.58, 95% confidence interval (CI) 0.46 to 0.72, *p* < 0.001) [10]. Similarly, the PALOMA-3 trial studied the combination of palbociclib and fulvestrant, resulting in a median PFS of 9.5 months compared with 4.6 months in the placebo-fulvestrant group (HR 0.46, 95% CI 0.36–0.59, *p* < 0.0001) [11]. Due to these substantial benefits, the National Comprehensive Cancer Network (NCCN) recommends CDK4/6 inhibitors as first-line therapy in HR-positive, HER2-negative metastatic breast cancer [12,13].

**Figure 1:**
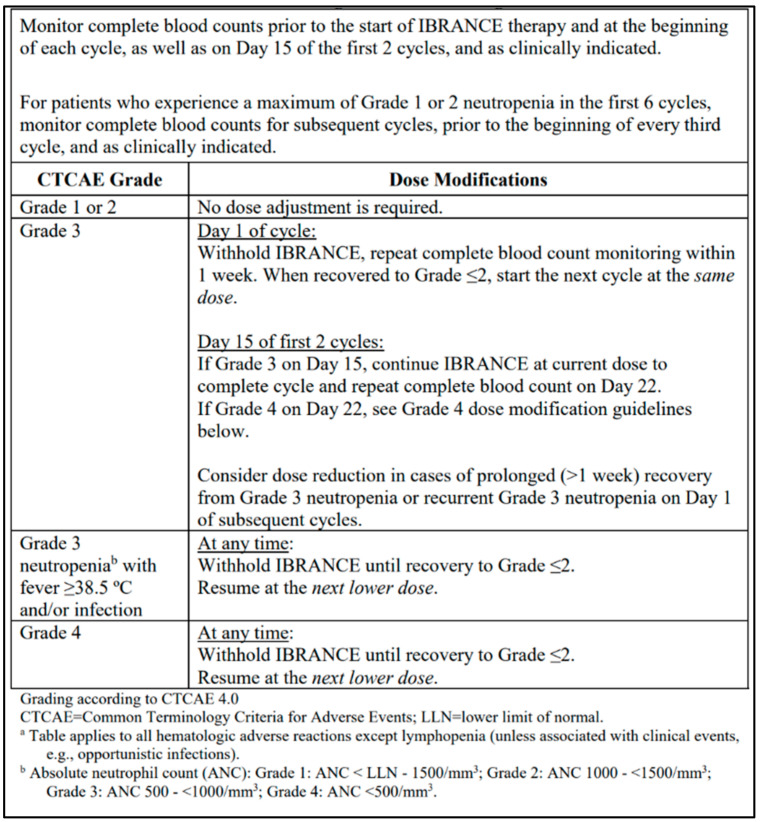
Dosing modifications and management of hematologic toxicities, reprinted from the Ibrance (palbociclib) manufacturer product monograph [15].

Various side effects have been associated with CDK4/6 inhibitors, including hematological toxicities, gastrointestinal effects, and cutaneous adverse reactions [14,15]. Neutropenia is the most common adverse effect of palbociclib and is managed through dose reductions, dose interruptions, or cycle delays [15]. Unlike systemic chemotherapy, neutropenia associated with palbociclib is rapidly reversible due to its cytostatic rather than cytotoxic effects on neutrophil precursors and rarely results in severe complications [16]. Palbociclib is conventionally dosed orally at 125 mg daily for 21 days, followed by a 7-day break for a total 28-day cycle. A complete blood count is recommended on days 1 and 15 for the first two cycles, and dose modifications are performed based on the manufacturer’s monograph recommendations (Figure 1) [15].

In real-world settings, oncologists have deviated from conventional monograph dosing recommendations and varied their prescribing strategies in attempts to mitigate neutropenic effects, hoping to maintain palbociclib tolerability and extend overall treatment duration. Recent studies have reported real-world outcomes and described dosing modifications trends [17,18,19,20,21,22,23,24,25,26,27]. However, to the best of our knowledge, this is the first real-world study assessing the direct impact of palbociclib off-monograph dosing modifications on PFS. With the increased adoption of alternative dosing strategies in practice, the effect on PFS of patients is unknown.

The aim of our study was to identify unique alternative dosing strategies of palbociclib prescribed by oncologists in a real-world setting and assess their impact on PFS in patients with HR-positive, HER2-negative metastatic breast cancer.

## 2. Materials and Methods

### 2.1. Study Design, Location, and Ethics

This was a retrospective, observational chart review of all patients at Trillium Health Partners (Credit Valley Hospital and Queensway Health Centre) treated with palbociclib in combination with letrozole or fulvestrant with a start date between June 2016 and December 2019 and followed until 30 April 2020. The Carlo Fidani Regional Cancer Centre located at the Credit Valley Hospital serves as a regional cancer centre, and the Queensway Health Centre is an academically affiliated health centre with a daytime ambulatory oncology clinic. Both sites are located within the Greater Toronto Area in Ontario, Canada.

Although palbociclib was introduced in our clinics in 2015 following Health Canada approval, we chose to collect data from June 2016 onwards when alternative dosing strategies were emerging.

This study was approved by the Research Ethics Board at Trillium Health Partners (ID #981) and operated in compliance with the Tri-Council Policy Statement, ICH GCP Guidelines, PHIPA, and Part C, Division 5 of the Health Canada Food and Drug Regulations.

### 2.2. Study Population

All female patients equal to or greater than 18 years of age diagnosed with HR-positive, HER2-negative metastatic breast cancer who received their first dose of palbociclib in combination with letrozole or fulvestrant between June 2016 to December 2018 were eligible. Exclusion criteria included patients who received one cycle or less of palbociclib or received prior CDK inhibitors, everolimus, or PI3K/mTOR pathway inhibitors. Patients who had no measurable metastatic disease, received previous chemotherapy or systemic therapy in the metastatic setting, discontinued palbociclib due to another adverse effect other than neutropenia, or did not continue on the assigned hormonal therapy were excluded.

### 2.3. Study Definitions

Progression-free survival was defined as the time from palbociclib initiation until discontinuation of treatment due to documented progression of disease, death, or time to the next line of therapy due to disease progression as recorded in clinician notes, whichever occurred first. Progression status was determined through clinical assessment by the oncologist using the RECIST criteria [28,29]. Neutropenia and thrombocytopenia were graded using the National Cancer Institute Common Terminology Criteria for Adverse Effects (CTCAE) [30]. Dosing strategies were categorized as either on- or off-monograph recommendations, with off-monograph dosing strategies reported descriptively [15]. Deviations from the monograph dosing recommendations are subsequently referred to as alternative dosing strategies.

### 2.4. Data Collection and Statistical Analysis

All data were collected from patient charts via Meditech and OPIS software. The collected data include the clinical site of treatment (Credit Valley Hospital or Queensway Health Centre), patient age, previous systemic and adjuvant treatments, prior endocrine therapy received, sites of metastatic disease, and Eastern Cooperative Oncology Group (ECOG) performance status at the time of palbociclib initiation. The information collected pertaining to palbociclib treatment includes date of treatment initiation, documented date of disease progression, death, or initiation of the next line of therapy due to progression, starting dose of palbociclib at treatment initiation, description of alternative dosing modifications where applicable, and total cycles of palbociclib received. The maximum grade of neutropenia was also recorded.

Kaplan–Meier curves were generated using R Statistical Software (v4.0.2; R Core Team 2020, Vienna, Austria) for patients receiving palbociclib in combination with letrozole and fulvestrant [31]. The curves were additionally stratified by patients prescribed monograph dosing recommendations and alternative dosing strategies. The median time in months to PFS with 95% confidence intervals was also reported where sample size allowed.

The number of patients remaining at risk of progression were reported at 6-month, 12-month, 15-month, and 18-month timepoints for patients receiving palbociclib in combination with letrozole and fulvestrant, and stratified according to the alternative dosing strategy prescribed. Differences between subgroups of dosing strategies were reported descriptively.

## 3. Results

### 3.1. Patient Demographics

**Figure 2:**
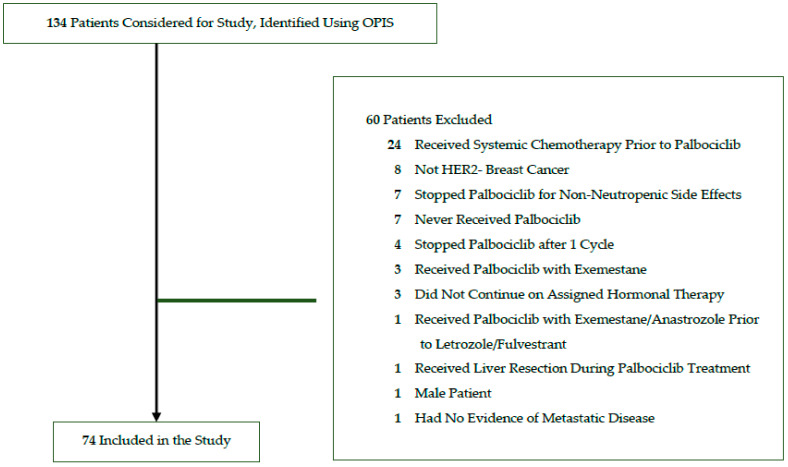
Study flow diagram for identified and included patients.

Between June 2016 to December 2019, a total of 134 patients were identified using OPIS who initiated palbociclib in combination with letrozole or fulvestrant at Trillium Health Partners. After applying exclusion criteria (see Section 2.2), 74 patients were included who initiated palbociclib in combination with letrozole (*n* = 63) or fulvestrant (*n* = 11) (Figure 2). Of these, 54 (73.0%) patients received care at the Credit Valley Hospital and the remaining 20 (27.0%) at Queensway Health Centre.

The mean age of all patients was 57.4 years (±standard deviation (SD) of 12.6); palbociclib and letrozole (P + L), 57.2 years (±12.5); and palbociclib and fulvestrant (P + F), 59.1 years (±14.0). Patients largely had an ECOG Performance Status of 0 (*n* = 19, 25.7%; P + L, *n* = 17, 27.0%; P + F, *n* = 2, 18.2%) or 1 (*n* = 49, 66.2%; P + L, *n* = 41, 65.1%; P + F, *n* = 8, 72.7%).

The most frequent site of metastatic disease was bone (*n* = 63, 85.1%; P + L, *n* = 56, 88.9%; P + F, *n* = 7, 63.6%), with more than one-third of patients having bone-only disease (*n* = 27, 36.5%; P + L, *n* = 25, 39.7%; P + F, *n* = 2, 18.2%). Other common sites included the lung (*n =* 18, 24.3%; P + L, *n* = 14, 22.2%; P + F, *n* = 4, 36.4%), pleura (*n* = 13, 17.6%; P + L, *n* = 11, 17.4%; P + F, *n* =2, 18.2%), and liver (*n* = 14, 18.9%; P + L, *n* = 8, 12.7%; P + F, *n* = 6, 54.5%).

While few letrozole patients received prior endocrine therapy, almost half of the fulvestrant patients received previous letrozole treatment (*n* = 5, 45.5%). Other previous adjuvant endocrine therapies received were tamoxifen (*n* = 32, 43.2%; P + L, *n* = 24, 38.1%; P + F, *n* = 8, 72.7%), anastrozole (*n* = 9, 12.2%; P + L, *n* = 7, 11.1%; P + F, *n* = 2, 18.2%), or exemestane (*n* = 5, 6.8%; P + L, *n* = 4, 6.3%; P + F, *n* = 1, 9.1%).

The mean cycles of palbociclib received were 17.9 (±8.9); P + L, 18.3 cycles (±8.7); and P + F, 15.4 cycles (±9.9).

Overall, 33 patients received dosing modifications (44.6%). Of these patients, more received alternative dosing modifications as compared to the monograph dosing recommendations (*n* = 29, 39.2%; P + L, *n* = 28, 44.4%; P + F, *n* = 1, 9.1%).

Full patient demographics are available in Table 1.

### 3.2. Overall Real-World Outcomes

**Figure 3:**
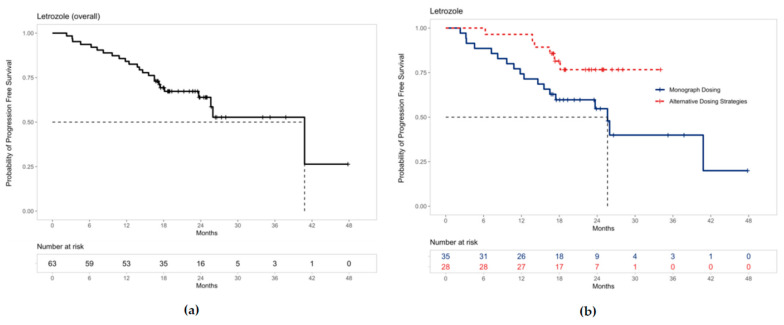
Progression-free survival for patients receiving palbociclib with letrozole: (**a**) The median PFS was 40.8 months (95% CI 25.6–not estimable) for all patients receiving palbociclib with letrozole. (**b**) The median PFS for patients receiving letrozole with palbociclib prescribed monograph dosing was 25.6 months (95% CI 16.5–not estimable). Median PFS could not be estimated for patients prescribed alternative dosing strategies due to the limited sample size. Note: ‘+’ points along the curves represent censored observations.

The median PFS for all patients on palbociclib with letrozole was 40.8 months (95% CI 25.6–not estimable) (Figure 3a). Separate Kaplan–Meier curves are shown to differentiate PFS for letrozole patients who were prescribed palbociclib based on monograph dosing versus alternative dosing strategies (Figure 3b). The median PFS for these patients receiving monograph dosing of palbociclib with letrozole was 25.6 months (95% CI 16.5–not estimable). Median PFS could not be estimated for patients prescribed palbociclib using alternative dosing strategies with letrozole due to the limited sample size.

**Figure 4:**
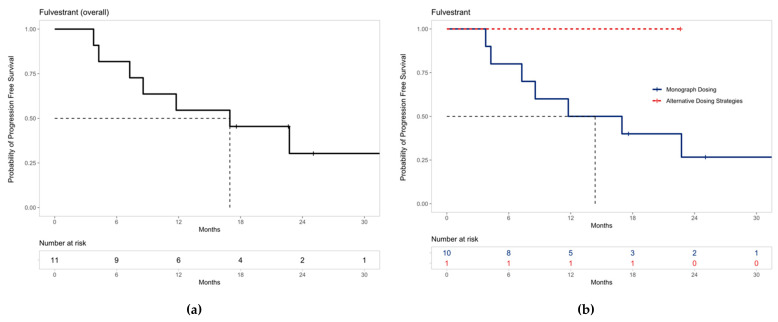
Progression-free survival for patients receiving palbociclib with fulvestrant: (**a**) The median PFS was 16.97 months (95% CI 8.57–not estimable) for all patients receiving palbociclib with fulvestrant. (**b**) The median PFS for patients receiving fulvestrant with palbociclib prescribed monograph dosing was 14.4 months (95% CI 7.27–not estimable). Median PFS could not be estimated for patients prescribed alternative dosing strategies due to the limited sample size. Note: ‘+’ points along the curves represent censored observations.

Similarly, the median PFS for all patients on palbociclib with fulvestrant was 16.97 months (95% CI 8.57–not estimable) (Figure 4a), and PFS is shown both for patients who were prescribed palbociclib based on monograph dosing and alternative dosing strategies (Figure 4b). The median PFS for patients receiving fulvestrant with palbociclib prescribed monograph dosing was 14.4 months (95% CI 7.27–not estimable). The median PFS could not be estimated for patients prescribed palbociclib using alternative dosing strategies with fulvestrant due to the limited sample size.

During treatment, 66.7% (*n* = 42) of the patients receiving palbociclib and letrozole experienced grade 3 neutropenia, and 6.3% (*n* = 4) experienced grade 4 neutropenia (see Table 2). For patients receiving palbociclib and fulvestrant, 45.5% (*n =* 5) of the patients experienced grade 3 neutropenia and 9.1% (*n* = 1) experienced grade 4 neutropenia (Table 2).

### 3.3. Alternative Dosing Strategies and Progression-Free Survival

Overall, 28 of 63 patients (44.4%) in the letrozole group and 1 of 11 (9.1%) patients in the fulvestrant group received alternative dosing modifications outside of the monograph recommendations. We identified seven unique alternative dosing strategies prescribed by oncologists at Trillium Health Partners (Table 3). Of these subgroups, the most frequent strategy was prescribing palbociclib for three weeks (21 days), followed by a two-week break as opposed to one week as per the monograph recommendations (*n* = 8; Table 3). This schedule was observed only in patients receiving palbociclib with letrozole. The second most common dosing strategy was a dose reduction of palbociclib, despite only experiencing one episode of grade 3 neutropenia, and this was observed in both letrozole (*n* = 6) and fulvestrant patients (*n* = 1) (Table 3).

For the group of letrozole patients treated with palbociclib prescribed three weeks on, two weeks off (*n* = 8), the number of patients remaining at risk of progression at 6, 12, 15, and 18 months were 8, 7, 6, and 4, respectively. The number of patients at risk of progression for all other alternative dosing strategies are detailed in Table 3. Many of these patients neither progressed nor were censored by the 18-month timepoint.

Of the eleven patients who received palbociclib in combination with fulvestrant, one patient received an alternative dosing strategy—a dose reduction of palbociclib despite only one episode of grade 3 neutropenia (Table 3). This patient continued to be observed even after 18 months of follow-up.

## 4. Discussion

### 4.1. Study Results in the Context of Existing Literature

The findings of this study contribute to the growing literature of real-world evidence with palbociclib from a Canadian context [17,18,32]. To the best of our knowledge, this is one of the first studies of its kind designed to investigate the effect of alternative dosing strategies on progression-free survival.

Our clinic population was comparable to other real-world studies, namely, the IRIS study and its Canadian cohort [17,21], as well as the landmark PALOMA randomized trials [10,11]. Our population was marginally younger (median 57.5 years, range 33–85) compared to the Canadian IRIS study (median 62 years, range 36–88) and the PALOMA trials (PALOMA-2 median 62 years, range 30–89 for the letrozole group and 61 years, range 28–88 for the placebo group; PALOMA-3 median 57 years, range 30–88 for the fulvestrant group and 56 years, range 29–80 for the placebo group). The majority of our patients were clinically stable with low symptom burden reflected by ECOG score of 0 to 1 and mainly bone metastatic disease, which is consistent with the IRIS and PALOMA trials.

The mean treatment cycles of palbociclib received were 17.9 cycles for all patients, with a longer mean of 18.3 cycles for letrozole and 15.4 cycles for fulvestrant. Although cycle counts do not capture prolongation of therapy due to withholding doses and delayed resumption of therapy, each cycle coarsely reflects a standard 28-day treatment course with periodic variations. Therefore, our follow-up was comparable with other real-world literature [17].

The median PFS observed in all our patients receiving palbociclib with letrozole (40.8 months, 95% CI 25.6–not estimable) is greater than real-world results such as the Canadian cohort from the IRIS study (20.2 months) and the PALOMA-2 trial (24.8 months, 95% CI 22.1–not estimable) [10,17]. Notably, the median PFS for our patients receiving letrozole with palbociclib prescribed monograph dosing is more comparable at 25.6 months (95% CI 16.5–not estimable).

Similarly, the median PFS observed for all patients receiving palbociclib with fulvestrant was 16.97 months (95% CI 8.57–not estimable), which is greater than other real-world trials [19] and the PALOMA-3 trial (9.5 months, 95% CI 9.2–11.0) [11]. Again, the median PFS for our patients receiving fulvestrant with palbociclib prescribed monograph dosing is more comparable at 14.4 months (95% CI 7.27–not estimable).

The differences in the observed median PFS for both our letrozole and fulvestrant patients compared to that in the existing literature is likely due to the short observational period and limited sample size. Additional studies within our clinic are required to confirm these findings.

Overall, our results with both letrozole and fulvestrant suggest a substantial benefit of palbociclib on PFS in women with HR-positive, HER2−negative metastatic breast cancer in a Canadian context, as observed in the existing literature [17,18,19,20,21,22,23,24,25,26,27].

### 4.2. Implications of Alternative Dosing Strategies on Efficacy and Safety Outcomes

A vast majority of patients (29 of the 33; 87.9%) who received dose modifications did so in a manner outside of monograph recommendations. Growing clinician familiarity with CDK4/6 inhibitors over time led to the emergence of alternative dosing strategies, resulting in the shorter observational time period for patients prescribed alternative dosing strategies compared to monograph dosing (Figure 3b and Figure 4b). The need for dose adjustments has also been commonly observed in other real-world literature and reflects the widespread approach of prescribers to circumvent palbociclib-associated neutropenia [17,19,20,21,23,25].

For palbociclib and letrozole specifically, the PFS curves for monograph dosing and alternative dosing strategies appear to diverge early in treatment and remain distinct over time (Figure 3b). This suggestive benefit in PFS may be the result of alternative dosing strategies mitigating neutropenic toxicities, increasing palbociclib tolerance, and prolonging treatment duration. These findings are exploratory yet novel, and further large-scale studies are warranted to confirm this result.

Notably, this study identified seven replicable alternative dosing strategies prescribed by oncologists at Trillium Health Partners. The most common dosing strategy was to prescribe palbociclib with letrozole for three weeks on, two weeks off (*n* = 8) (Table 3). Even after 18 months, half of these patients continued to be observed and remained progression-free in our study. Moreover, a high number of patients continued to be observed by 18 months with all seven alternative dosing strategies.

For our letrozole patients, more patients experienced grade 3 neutropenia and less grade 4 neutropenia when compared to the PALOMA-2 trial (Table 2) [10]. Grade 3 and 4 neutropenia rates for our fulvestrant patients were almost identical to those in PALOMA-3 [11]. Although this may reflect differences between a robust randomized trial and real-world practice, we believe the reduction in the severity of neutropenia may also be attributed to these alternative dosing strategies. For example, by extending the break duration of palbociclib to two weeks, neutrophil counts recover adequately and patients may resume treatment at the same dose level. Thus, this strategy exploits the cytostatic nature of neutropenia caused by CDK4/6 inhibition and is yet another advantage of the mechanisms targeting cell cycle arrest as opposed to traditional chemotherapy [16].

We conclude that these alternative dosing strategies identified at our clinics appear to have promising PFS benefits while mitigating potential neutropenia related to palbociclib therapy, prolonging treatment tolerability and overall adherence. Anecdotally, we also observed other benefits such as patients achieving stable doses more quickly, requiring less frequent bloodwork and clinic visits, consequentially improving the cost effectiveness of care and patient satisfaction. Recognizing this is a retrospective study with a short observational period and limited sample size, future large-scale studies are strongly recommended to confirm statistical non-inferiority or superiority of these alternative dosing strategies as compared to standard monograph dosing.

### 4.3. Limitations of the Study Design

We recognize the findings of our study are limited by the small sample size and short follow-up duration. Due to the severity of the COVID-19 pandemic with provincial restrictions implemented in April 2020, we selected an earlier cut-off date of 30 April 2020 to avoid the unknown impact of practice changes related to the pandemic. Such changes include increased remote clinic appointments and decreased frequency of bloodwork. We acknowledge that, in ideal circumstances, a follow-up duration at minimum would have been 24 months for all patients to match the average PFS of 24.8 months as seen in the PALOMA-2 landmark trial.

Secondly, our study descriptively compared results with no formal statistical analysis. Despite combining data across our institution, the recent introduction of palbociclib in Canada prevented the feasibility of recruiting a sufficient sample and statistical comparison at this time.

Finally, the population we studied focused on clinically stable patients with mainly an ECOG score of 0 or 1 and bone-only metastatic disease and excluded patients who received prior chemotherapy. Caution should be taken when extrapolating these results to patients who do not fit these characteristics. Overall, we cannot rule out baseline characteristics such as ECOG performance status or sites of metastases as potential confounding factors due to the limited sample size.

### 4.4. Ongoing Research Surrounding Alternative Prescribing Schedules

At present, alternative prescribing schedules are being explored in phase 2 trials, specifically trialing palbociclib 100 mg in continuous daily dosing [33] and palbociclib given five days on, two days off every seven days [34]. As more efficacy and safety results become available, future research should be directed towards identifying which alternative dosing strategy is best suited for each patient population. Furthermore, additional studies may explore whether comparable PFS is achieved when prescribing alternative dosing strategies in men, or whether these findings may be extrapolated across other CDK 4/6 inhibitors as a class effect.

## 5. Conclusions

The median progression-free survival for all patients receiving palbociclib and letrozole was 40.8 months (95% CI 25.6–not estimable) and 16.97 months (95% CI 8.57–not estimable) for palbociclib and fulvestrant. We identified seven unique alternative dosing strategies of palbociclib prescribed by oncologists at Trillium Health Partners. The Kaplan–Meier curves for PFS in patients receiving letrozole and palbociclib prescribed alternative dosing strategies appear to diverge from monograph dosing early in the treatment, while the frequency of grade 4 neutropenia was lower in the letrozole patients. Many patients prescribed alternative dosing strategies continued to be observed even by the 18-month timepoint. We conclude that alternative dosing strategies such as prescribing palbociclib for three weeks on, two weeks off may achieve comparable disease control while mitigating neutropenic toxicities, improving treatment tolerability and adherence. Given the small sample size in this study, our findings are exploratory and further large-scale studies are required to confirm these results for future clinical adoption.

## Figures and Tables

**Table 1:** Patient demographics and clinical characteristics.

Characteristics	Overall (*n* = 74)	Palbociclib with Letrozole (*n* = 63)	Palbociclib with Fulvestrant (*n* = 11)
Treatment Site*—n* (%)			
Credit Valley Hospital	54 (73.0%)	46 (73.0%)	8 (72.7%)
Queensway Health Centre	20 (27.0%)	17 (27.0%)	3 (27.3%)
Age			
Mean (SD)	57.4 (12.6)	57.2 (12.5)	59.1 (14.0)
Median (range)	57.5 (33–85)	55 (34–85)	60 (33–77)
<65—*n* (%)	52 (70.3%)	44 (69.8%)	8 (72.7%)
≥65—*n* (%)	22 (29.7%)	19 (30.2%)	3 (27.3%)
ECOG Performance Status—*n* (%)			
0	19 (25.7%)	17 (27.0%)	2 (18.2%)
1	49 (66.2%)	41 (65.1%)	8 (72.7%)
2	5 (6.8%)	5 (7.9%)	0 (0%)
3	1 (1.4%)	0 (0%)	1 (9.1%)
Disease Site			
Bone	63 (85.1%)	56 (88.9%)	7 (63.6%)
Bone only	27 (36.5%)	25 (39.7%)	2 (18.2%)
Lung	18 (24.3%)	14 (22.2%)	4 (36.4%)
Pleura	13 (17.6%)	11 (17.4%)	2 (18.2%)
Liver	14 (18.9%)	8 (12.7%)	6 (54.5%)
CNS	2 (2.7%)	1 (1.6%)	1 (9.1%)
Other	16 (21.6%)	14 (22.2%)	2 (18.2%)
Received prior adjuvant or neoadjuvant endocrine therapy	13 (17.6%)	7 (11.1%)	6 (54.5%)
Received prior endocrine therapy in the metastatic setting			
Anastrozole	1 (1.4%)	0 (0%)	1 (9.1%)
Exemestane	5 (6.8%)	3 (4.8%)	2 (18.2%)
Letrozole	5 (6.8%)	0 (0%)	5 (45.5%)
Tamoxifen	6 (8.1%)	5 (7.9%)	1 (9.1%)
Received prior adjuvant therapy			
Anastrozole	9 (12.2%)	7 (11.1%)	2 (18.2%)
Exemestane	5 (6.8%)	4 (6.3%)	1 (9.1%)
Letrozole	2 (2.7%)	1 (1.6%)	1 (9.1%)
Tamoxifen	32 (43.2%)	24 (38.1%)	8 (72.7%)
Average cycles of palbociclib received			
Mean (SD)	17.9 (8.9)	18.3 (8.7)	15.4 (9.9)
Median (range)	17 (2–48)	17 (2–48)	15 (3–34)
Any dose modifications received (monograph or unique)	33 (44.6%)	30 (47.6%)	3 (27.3%)
Monograph dose reductions	4 (5.4%)	2 (3.2%)	2 (18.2%)
Alternative dosing strategies	29 (39.2%)	28 (44.4%)	1 (9.1%)

**Table 2:** Highest grade of neutropenia experienced by patients receiving palbociclib in combination with letrozole or fulvestrant.

Treatment Group	Grade 3 *n* (%)	Grade 4 *n* (%)	Grade 3 and 4 *n* (%)
Letrozole (*n* = 63)	42 (66.7%)	4 (6.3%)	46 (73%)
Fulvestrant (*n* = 11)	5 (45.5%)	1 (9.1%)	6 (54.5%)

**Table 3:** Clinic patients remaining at risk of progression at 6, 12, 15, and 18 months receiving palbociclib in combination with letrozole or fulvestrant stratified by dosing strategy.

Palbociclib Dosing Strategy	Patients (*n*)	6 Months (*n)*	12 Months (*n)*	15 Months (*n)*	18 Months (*n)*
Letrozole (Overall)	63	59	53	49	35
Monograph dosing	35	31	26	24	18
Alternative Dosing Strategies					
Palbociclib prescribed 3 weeks on, 2 weeks off	8	8	7	6	4
Palbociclib dose decreased for only one episode of ANC < 1.0 × 10^9^/L	6	6	6	5	4
Lowered palbociclib dose despite ANC > 1.0 × 10^9^/L	5	5	5	5	4
Remained on palbociclib 75 mg despite recurrent grade 3 neutropenia	5	5	5	5	2
Decreased palbociclib from 125 mg to 75 mg for grade 3 neutropenia	2	2	2	2	2
Palbociclib initiated at 100 mg daily	1	1	1	1	0
Palbociclib prescribed 2 weeks on, 2 weeks off	1	1	1	1	1
Fulvestrant (Overall)	11	9	6	6	4
Monograph dosing	10	8	5	5	3
Alternative Dosing Strategies					
Palbociclib dose decreased for only one episode of ANC < 1.0 × 10^9^/L	1	1	1	1	1

ANC = absolute neutrophil count. Note: ‘Months’ are 30-day intervals.

## Data Availability

The data presented in this study are available on request from the corresponding author.

## References

[1] Ferlay J., Soerjomataram I., Dikshit R., Eser S., Mathers C., Rebelo M., Parkin D.M., Forman D., Bray F. (2015). Cancer Incidence and Mortality Worldwide: Sources, Methods and Major Patterns in GLOBOCAN 2012. Int. J. Cancer.

[2] Torre L.A., Islami F., Siegel R.L., Ward E.M., Jemal A. (2017). Global Cancer in Women: Burden and Trends. Cancer Epidemiol. Biomark. Prev. Publ. Am. Assoc. Cancer Res. Cosponsored Am. Soc. Prev. Oncol..

[3] Harbeck N., Gnant M. (2017). Breast Cancer. Lancet.

[4] Perou C.M., Sørlie T., Eisen M.B., van de Rijn M., Jeffrey S.S., Rees C.A., Pollack J.R., Ross D.T., Johnsen H., Akslen L.A. (2000). Molecular Portraits of Human Breast Tumours. Nature.

[5] Sørlie T., Perou C.M., Tibshirani R., Aas T., Geisler S., Johnsen H., Hastie T., Eisen M.B., van de Rijn M., Jeffrey S.S. (2001). Gene Expression Patterns of Breast Carcinomas Distinguish Tumor Subclasses with Clinical Implications. Proc. Natl. Acad. Sci. USA.

[6] Howlader N., Altekruse S.F., Li C.I., Chen V.W., Clarke C.A., Ries L.A.G., Cronin K.A. (2014). US Incidence of Breast Cancer Subtypes Defined by Joint Hormone Receptor and HER2 Status. JNCI J. Natl. Cancer Inst..

[7] Rugo H.S., Rumble R.B., Macrae E., Barton D.L., Connolly H.K., Dickler M.N., Fallowfield L., Fowble B., Ingle J.N., Jahanzeb M. (2016). Endocrine Therapy for Hormone Receptor-Positive Metastatic Breast Cancer: American Society of Clinical Oncology Guideline. J. Clin. Oncol. Off. J. Am. Soc. Clin. Oncol..

[8] de Groot A.F., Kuijpers C.J., Kroep J.R. (2017). CDK4/6 Inhibition in Early and Metastatic Breast Cancer: A Review. Cancer Treat. Rev..

[9] O’Leary B., Finn R.S., Turner N.C. (2016). Treating Cancer with Selective CDK4/6 Inhibitors. Nat. Rev. Clin. Oncol..

[10] Finn R.S., Martin M., Rugo H.S., Jones S., Im S.-A., Gelmon K., Harbeck N., Lipatov O.N., Walshe J.M., Moulder S. (2016). Palbociclib and Letrozole in Advanced Breast Cancer. N. Engl. J. Med..

[11] Cristofanilli M., Turner N.C., Bondarenko I., Ro J., Im S.-A., Masuda N., Colleoni M., DeMichele A., Loi S., Verma S. (2016). Fulvestrant plus Palbociclib versus Fulvestrant plus Placebo for Treatment of Hormone-Receptor-Positive, HER2-Negative Metastatic Breast Cancer That Progressed on Previous Endocrine Therapy (PALOMA-3): Final Analysis of the Multicentre, Double-Blind, Phase 3 Randomised Controlled Trial. Lancet Oncol..

[12] Gradishar W.J., Anderson B.O., Balassanian R., Blair S.L., Burstein H.J., Cyr A., Elias A.D., Farrar W.B., Forero A., Giordano S.H. (2017). NCCN Guidelines Insights: Breast Cancer, Version 1.2017. J. Natl. Compr. Cancer Netw. JNCCN.

[13] Giordano S.H., Elias A.D., Gradishar W.J. (2018). NCCN Guidelines Updates: Breast Cancer. J. Natl. Compr. Cancer Netw. JNCCN.

[14] Silvestri M., Cristaudo A., Morrone A., Messina C., Bennardo L., Nisticò S.P., Mariano M., Cameli N. (2021). Emerging Skin Toxicities in Patients with Breast Cancer Treated with New Cyclin-Dependent Kinase 4/6 Inhibitors: A Systematic Review. Drug Saf..

[15] (2020). Pfizer Canada ULC Ibrance Product Monograph. https://www.pfizer.ca/sites/default/files/202107/Ibrance_PM_EN_243405_15-Jul-2021.pdf.

[16] Spring L.M., Zangardi M.L., Moy B., Bardia A. (2017). Clinical Management of Potential Toxicities and Drug Interactions Related to Cyclin-Dependent Kinase 4/6 Inhibitors in Breast Cancer: Practical Considerations and Recommendations. Oncologist.

[17] Mycock K., Zhan L., Taylor-Stokes G., Milligan G., Mitra D. (2021). Real-World Palbociclib Use in HR+/HER2- Advanced Breast Cancer in Canada: The IRIS Study. Curr. Oncol. Tor. Ont.

[18] Amaro C.P., Batra A., Lupichuk S. (2021). First-Line Treatment with a Cyclin-Dependent Kinase 4/6 Inhibitor Plus an Aromatase Inhibitor for Metastatic Breast Cancer in Alberta. Curr. Oncol..

[19] Bui T.B.V., Burgers D.M., Agterof M.J., van de Garde E.M. (2019). Real-World Effectiveness of Palbociclib Versus Clinical Trial Results in Patients with Advanced/Metastatic Breast Cancer That Progressed on Previous Endocrine Therapy. Breast Cancer Basic Clin. Res..

[20] Wilkie J., Schickli M.A., Berger M.J., Lustberg M., Reinbolt R., Noonan A., Ramaswamy B., Sardesai S., VanDeusen J., Wesolowski R. (2020). Progression-Free Survival for Real-World Use of Palbociclib in Hormone Receptor-Positive Metastatic Breast Cancer. Clin. Breast Cancer.

[21] Taylor-Stokes G., Mitra D., Waller J., Gibson K., Milligan G., Iyer S. (2019). Treatment Patterns and Clinical Outcomes among Patients Receiving Palbociclib in Combination with an Aromatase Inhibitor or Fulvestrant for HR+/HER2-Negative Advanced/Metastatic Breast Cancer in Real-World Settings in the US: Results from the IRIS Study. Breast Edinb. Scotl..

[22] Varella L., Eziokwu A.S., Jia X., Kruse M., Moore H.C.F., Budd G.T., Abraham J., Montero A.J. (2019). Real-World Clinical Outcomes and Toxicity in Metastatic Breast Cancer Patients Treated with Palbociclib and Endocrine Therapy. Breast Cancer Res. Treat..

[23] Watson G.A., Deac O., Aslam R., O’Dwyer R., Tierney A., Sukor S., Kennedy J. (2019). Real-World Experience of Palbociclib-Induced Adverse Events and Compliance With Complete Blood Count Monitoring in Women With Hormone Receptor-Positive/HER2-Negative Metastatic Breast Cancer. Clin. Breast Cancer.

[24] Lee J., Park H.S., Won H.S., Yang J.H., Lee H.Y., Woo I.S., Shin K., Hong J.H., Yang Y.J., Chun S.H. (2021). Real-World Clinical Data of Palbociclib in Asian Metastatic Breast Cancer Patients: Experiences from Eight Institutions. Cancer Res. Treat..

[25] Harbeck N., Bartlett M., Spurden D., Hooper B., Zhan L., Rosta E., Cameron C., Mitra D., Zhou A. (2021). CDK4/6 Inhibitors in HR+/HER2- Advanced/Metastatic Breast Cancer: A Systematic Literature Review of Real-World Evidence Studies. Future Oncol..

[26] DeMichele A., Cristofanilli M., Brufsky A., Liu X., Mardekian J., McRoy L., Layman R.M., Emir B., Torres M.A., Rugo H.S. (2021). Comparative Effectiveness of First-Line Palbociclib plus Letrozole versus Letrozole Alone for HR+/HER2- Metastatic Breast Cancer in US Real-World Clinical Practice. Breast Cancer Res. BCR.

[27] Fernández-Cuerva C., Valencia J.C.D.R., Bermejo R.T. (2022). Effectiveness and Safety of Palbociclib plus Endocrine Therapy in Hormone Receptor-Positive, HER2-Negative Metastatic Breast Cancer: Real-World Results. Can. J. Hosp. Pharm..

[28] Eisenhauer E.A., Therasse P., Bogaerts J., Schwartz L.H., Sargent D., Ford R., Dancey J., Arbuck S., Gwyther S., Mooney M. (2009). New Response Evaluation Criteria in Solid Tumours: Revised RECIST Guideline (Version 1.1). Eur. J. Cancer.

[29] Schwartz L.H., Litière S., de Vries E., Ford R., Gwyther S., Mandrekar S., Shankar L., Bogaerts J., Chen A., Dancey J. (2016). RECIST 1.1-Update and Clarification: From the RECIST Committee. Eur. J. Cancer.

[30] (2017). Common Terminology Criteria for Adverse Events (CTCAE). https://ctep.cancer.gov/protocoldevelopment/electronic_applications/docs/ctcae_v5_quick_reference_8.5x11.pdf.

[31] R: The R Project for Statistical Computing. https://www.r-project.org/.

[32] Tripathy D., Blum J.L., Rocque G.B., Bardia A., Karuturi M.S., Cappelleri J.C., Liu Y., Zhang Z., Davis K.L., Wang Y. (2020). POLARIS: A Prospective, Multicenter, Noninterventional Study Assessing Palbociclib in Hormone Receptor-Positive Advanced Breast Cancer. Future Oncol..

[33] Parulekar W.R., Joy A.A., Gelmon K., Mates M., Desbiens C., Clemons M., Taylor S., Lemieux J., Bartlett J., Whelan T. (2019). Abstract PD1-10: Randomized Phase II Study Comparing Two Different Schedules of Palbociclib plus Second Line Endocrine Therapy in Women with Estrogen Receptor Positive, HER2 Negative Advanced/Metastatic Breast Cancer: CCTG MA38 (NCT02630693). Cancer Res..

[34] Krishnamurthy J., Luo J., Ademuyiwa F., Suresh R., Rigden C., Reardon T., Weilbaecher K., Frith A., Roshal A., Tandra P. (2020). Abstract P1-19-13: A Phase II Trial Assessing the Safety of an Alternative Dosing Schedule of Palbociclib (Palbo) in Hormone Receptor Positive (HR+), HER2 Negative (HER2-) Metastatic Breast Cancer (MBC): Alt Dose Palbo. Cancer Res..

